# Case Report: Sulcal artery infarction presenting as incomplete Brown-Séquard syndrome following spinal anesthesia in a 70-year-old female: a rare postoperative neurological complication

**DOI:** 10.3389/fradi.2025.1672382

**Published:** 2025-11-03

**Authors:** B. T. Kavya, Shweta Raviraj Poojary, Harsha Sundaramurthy

**Affiliations:** ^1^ Department of Radiodiagnosis, JSS Academy of Higher Education and Research, Mysore, India; ^2^ Department of Radiodiagnosis, Kasturba Medical College Mangalore, Manipal Academy of Higher Education, Manipal, India; ^3^ Department of Neurology, JSS Academy of Higher Education and Research, Mysore, India

**Keywords:** sulcal artery infarction, spinal cord stroke, Brown-Séquard syndrome, spinal anesthesia, hemicord lesion

## Abstract

Spinal cord infarction following neuraxial anesthesia is a rare but serious complication. We present the case of a 70-year-old female who developed acute onset of left lower limb weakness immediately following spinal anesthesia administered for total hip replacement. Clinical features were consistent with incomplete Brown-Séquard syndrome. MRI revealed a T2/STIR hyperintense lesion involving the left hemicord at the D12-L1 vertebral level, suggestive of sulcal artery infarction. MRI showed only age-related changes. After a structured physiotherapy program, the patient experienced significant functional improvement and was discharged with stable vitals. This case highlights the importance of early diagnosis and management of spinal cord infarction in the perioperative setting.

## Introduction

Spinal cord infarction is a rare neurological emergency that typically occurs in association with aortic or vascular procedures, hypotension, or embolic phenomena. It accounts for less than 1% of all strokes (1). Among these, sulcal artery infarction, a variant of anterior spinal artery syndrome, may present as hemicord involvement, sometimes resulting in incomplete Brown-Séquard syndrome. This clinical pattern involves ipsilateral motor weakness and proprioceptive loss with contralateral loss of pain and temperature sensation. Although spinal anesthesia is widely considered safe, rare instances of spinal cord ischemia have been reported (2–5). Here, we report a case of sulcal artery infarction occurring immediately after spinal anesthesia for orthopedic surgery, presenting with an incomplete Brown-Séquard syndrome.

**Figure 1:**
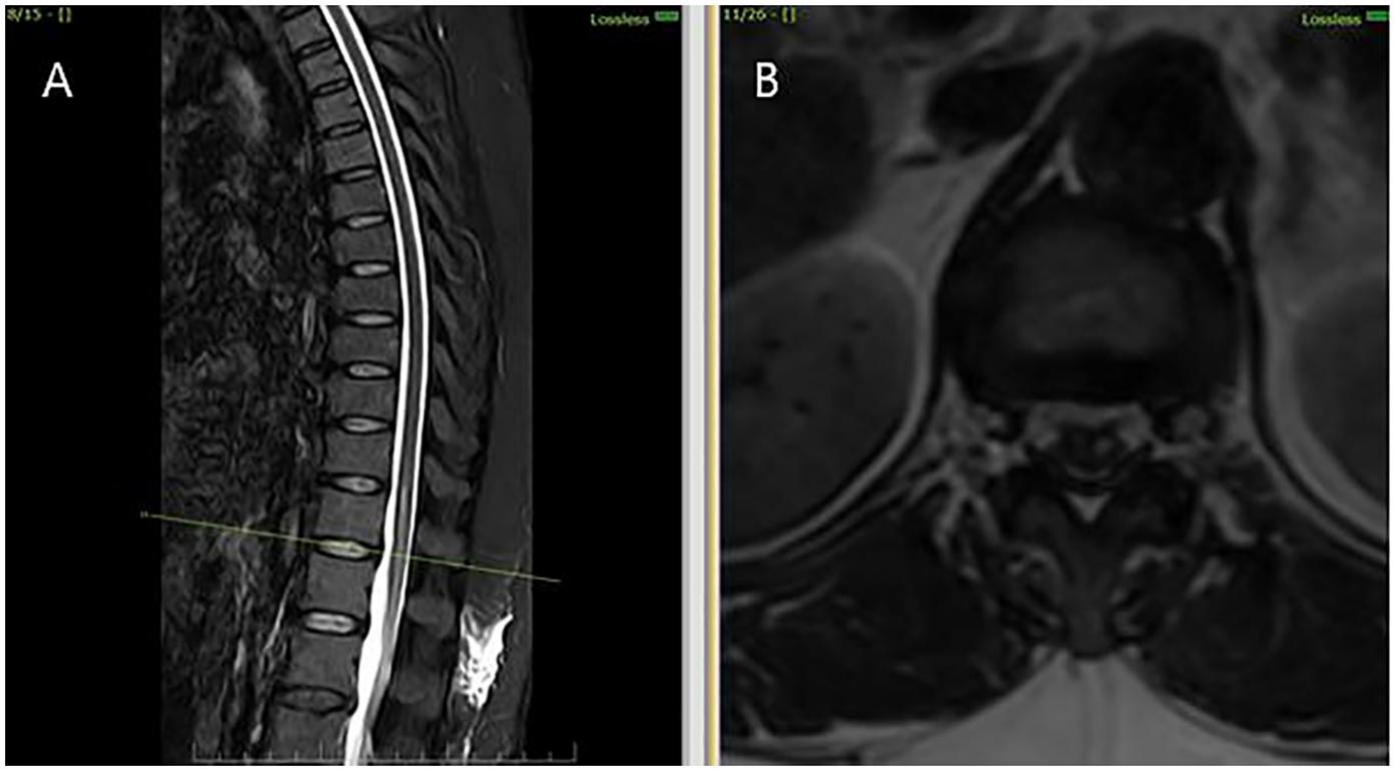
MRI thoracolumbar spine findings suggestive of sulcal artery infarction. **(A)** Sagittal T2-weighted MRI shows a linear hyperintense lesion in the anterior portion of the spinal cord at the D12 -L1 level, with no evidence of compressive pathology. **(B)** Axial T2-weighted MRI at the D12- L1 level demonstrates hyperintensity involving the anterior horn and intermediate zone of the left hemicord, consistent with sulcal artery territory infarction.

## Case-report

A 70-year-old female with a history of degenerative hip disease was scheduled for elective total hip replacement. Her medical history was otherwise unremarkable, with no known vascular risk factors such as diabetes, hypertension, or cardiac disease. She underwent spinal anaesthesia using a standard single-shot lumbar subarachnoid injection. The anesthetic used was Bupivacaine (Heavy) 0.5% 3 mL with Fentanyl 25 µg (0.5 mL), given as a single-shot injection. The level of anesthesia achieved was T6–T8. No complications were noted during the procedure, and intraoperative haemodynamics remained stable.

Immediately following surgery, the patient reported acute weakness in the left lower limb, accompanied by sensory deficits. She reported loss of touch and pain in the left lower limb and frequent micturition, but no urinary retention or bowel incontinence. Physical examination revealed motor power of 3/5 in the left lower limb. Sensory examination demonstrated contralateral (right-sided) loss of pain and temperature sensation below the level of the umbilicus, with preserved proprioception and vibration on the affected (left) side. Reflexes were brisk on the left, and no signs of meningeal irritation or bladder/bowel involvement were present.

There was no prior history of spinal interventions, trauma, or autoimmune disease.

### Diagnostic assessment

**Figure 2:**
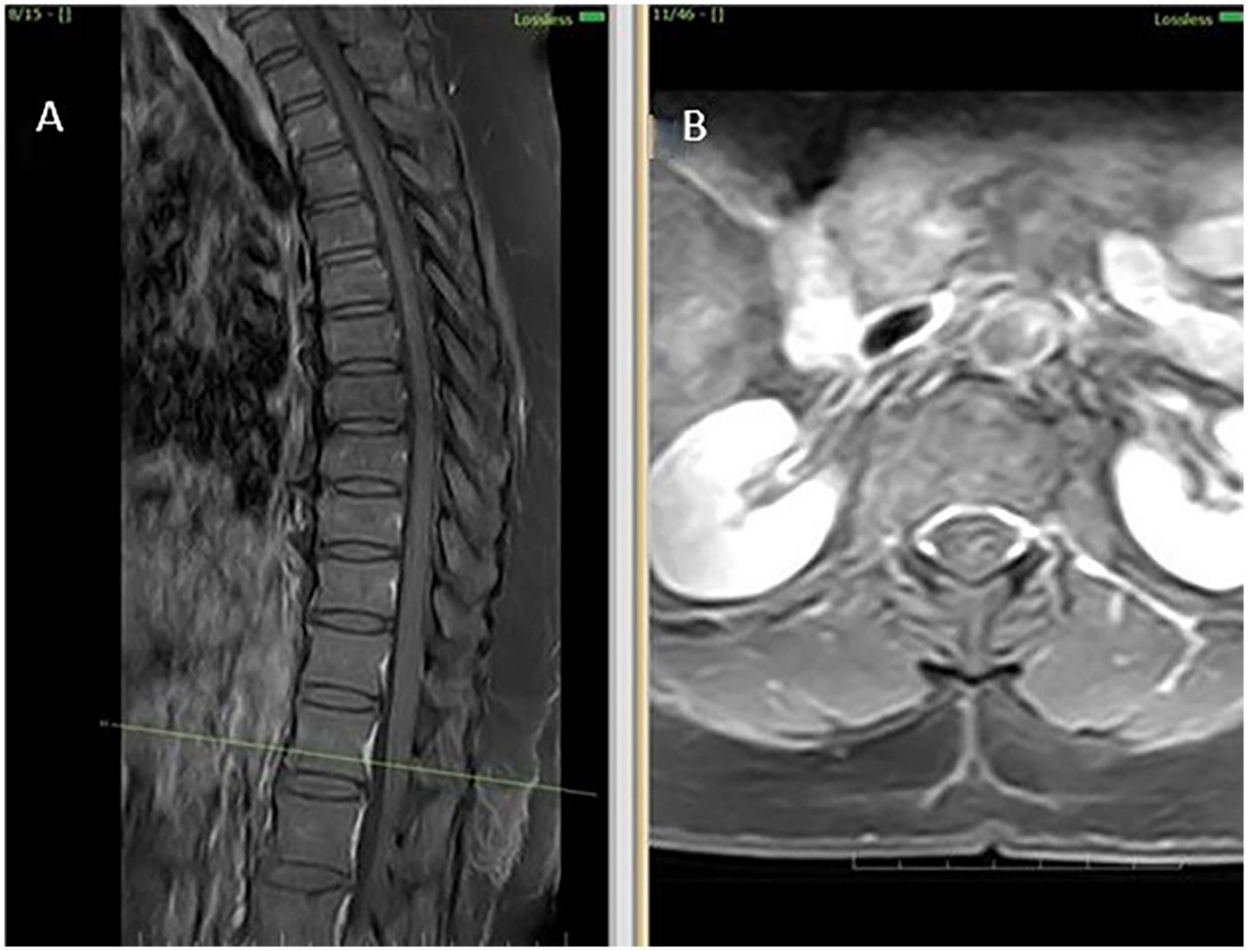
MRI thoracolumbar spine findings suggestive of sulcal artery infarction. **(A)** Sagittal post-contrast T1-weighted image reveals no contrast enhancement at the lesion site, ruling out inflammatory or neoplastic causes. **(B)** Axial post-contrast T1-weighted MRI confirms absence of mass effect or enhancement, supporting a non-compressive vascular etiology.

**Figure 3:**
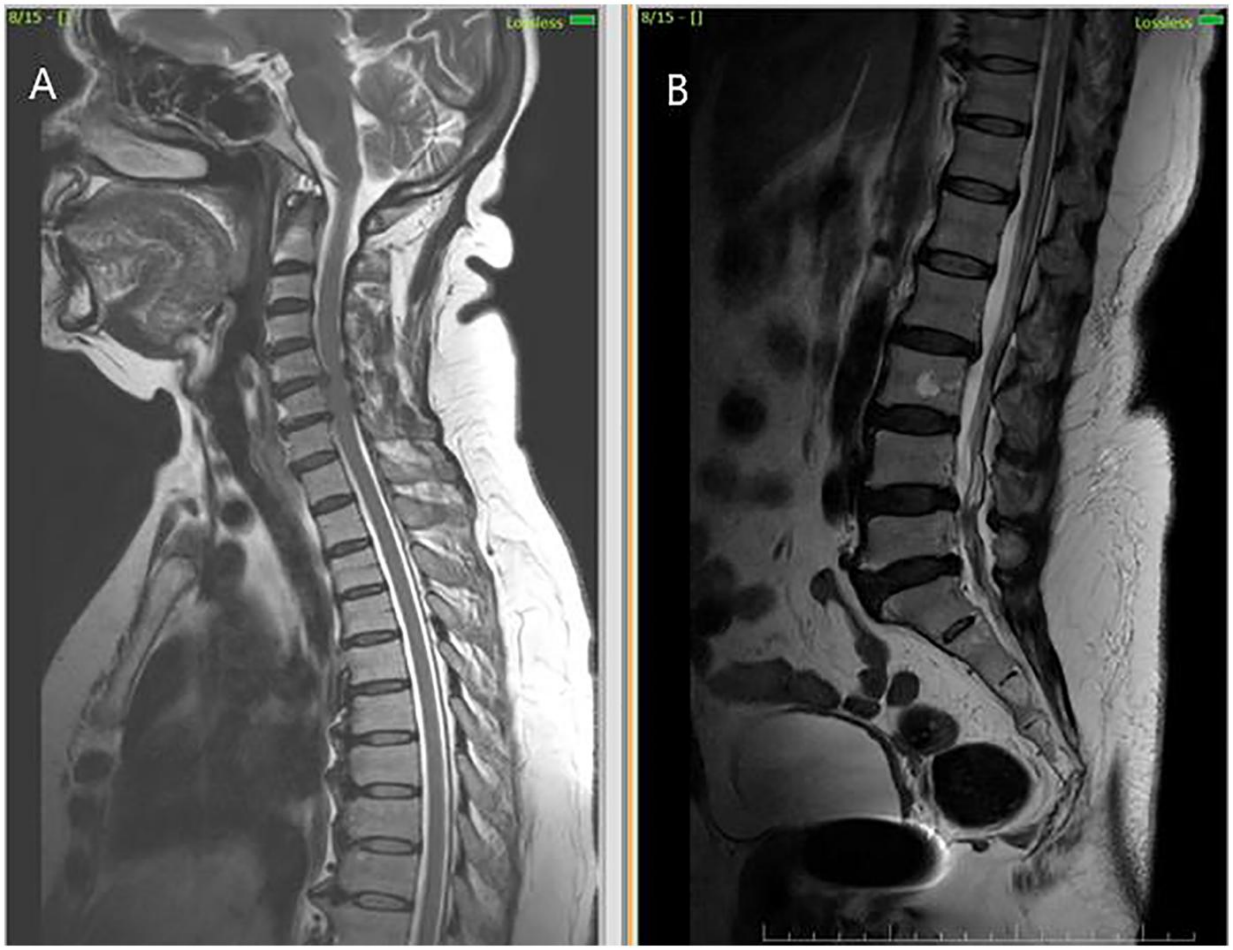
MRI cervical and lumbar spine findings suggestive of spondylosis. **(A)** Sagittal T2-weighted MRI shows cervical spondylosis with disc degenerative changes **(B)** Sagittal T2-weighted MRI shows lumbar spondylosis with disc degenerative changes.

Initial investigations (CBC, ESR, CRP, renal and liver function, echocardiography, ECG) were unremarkable. MRI thoracolumbar spine was performed 1 month and 28 days after symptom onset. MRI brain showed no signs of infarction or demyelination. MRI thoracolumbar spine demonstrated T2 and STIR hyperintensity involving the anterior horn and intermediate zone of the left hemicord at D12-L1, without contrast enhancement or mass effect. There was no evidence of cord compression, hematoma, or abscess (Figures 2A–D). MRI of the cervical and lumbar spine showed spondylotic changes with no significant cord involvement (Figures 3A,B). Poor-quality DWI was obtained, limiting diagnostic utility (acknowledged as a limitation). No follow-up MRI was performed.

Autoimmune and vascular evaluation including ANA, antiphospholipid antibodies, homocysteine, and coagulation profile revealed no abnormalities. The diagnosis of sulcal artery infarction was made based on clinical presentation and imaging findings. Given the absence of systemic or embolic sources, the infarct was attributed to idiopathic microvascular occlusion, possibly precipitated by peri-procedural microvascular hypoperfusion. No digital subtraction angiography (DSA) was performed, which remains a limitation in excluding occult vascular malformations.

### Therapeutic intervention and follow-up

The patient was admitted to a monitored care unit and managed conservatively. She received: Antiplatelet therapy

DVT prophylaxis

### Structured physiotherapy and neurorehabilitation

Reflexes remained brisk on the left side with an extensor plantar response. Over the course of three weeks, she demonstrated progressive improvement in motor function. By the fourth week, she was ambulatory with assistance. At discharge in the fifth week, she had regained substantial strength and was advised to continue outpatient rehabilitation.

## Discussion

Acute spinal cord syndromes pose a diagnostic challenge, especially when differentiating between infectious, inflammatory, neoplastic, and vascular aetiologies. Spinal cord infarction is rare, and infarction of the sulcal artery, a central branch of the anterior spinal artery supplying the medial portion of the hemicord, is even less frequently reported. When affected, it can lead to hemicord ischemia, presenting clinically as incomplete Brown-Séquard syndrome or its variants.

The clinical Algorithm in the below mentioned decision tree is particularly valuable in narrowing down the diagnosis based on symptomatology (Table 1).

**Table 1:** Clinical algorithm.

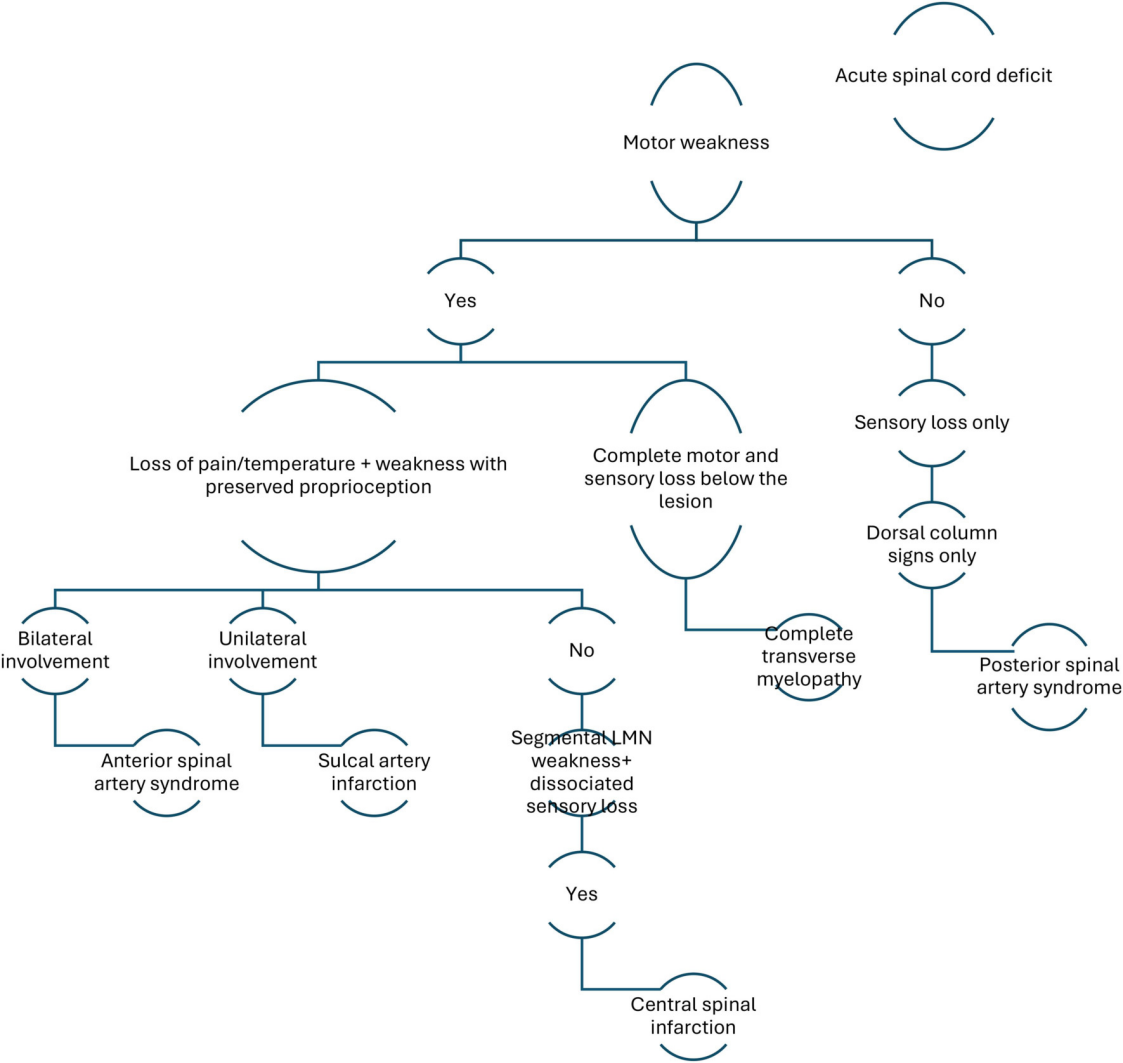	

In this case, the patient presented with an acute onset of unilateral motor weakness and loss of pain and temperature sensation, with relative preservation of proprioception. This specific neurological pattern localises to a hemicord involvement, pointing towards a vascular insult in the sulcal artery territory. The decision tree appropriately classifies this pattern under sulcal artery infarction, a less commonly recognised anterior spinal cord infarction subtype.

MRI findings further corroborated the clinical suspicion: there was T2 hyperintensity predominantly involving the anterior horn and intermediate zone of the left hemicord at D12- L1 vertebral level without enhancement or mass effect. In the absence of compressive or infectious causes, the radiologic diagnosis of non-traumatic spinal cord infarct is strongly supported.

Most sulcal artery infarctions described in the literature have involved the cervical or upper thoracic spinal cord6. For instance, Sharma et al. reported hemicord infarctions predominantly in elderly individuals, often with vascular comorbidities and typical localisation to the cervical cord7. Similarly, other case series and reviews have highlighted upper spinal cord involvement due to higher metabolic demands and susceptibility to hypotensive or embolic insults.8 In contrast, our case is unique in demonstrating sulcal artery infarction at the thoracolumbar junction (D12 -L1), a level rarely reported in published literature 6,9.

The temporal relationship of infarct onset immediately after spinal anaesthesia further distinguishes this case. While neuraxial anaesthesia is generally considered safe, rare complications such as spinal cord ischaemia have been reported, usually secondary to hypotension, vasospasm, or mechanical injury (2–5). In this case, there were no haemodynamic fluctuations, technical difficulties, or radiologic evidence of hematoma or compressive pathology. Nevertheless, the close procedural timing raises the possibility of anaesthesia-induced microvascular compromise, particularly affecting a sulcal artery. This mechanism, although speculative, remains plausible given the vascular fragility in elderly patients and the confined vascular supply of the anterior spinal system. This case reinforces the importance of considering vascular causes in acute myelopathy, especially when imaging localises a unilateral anterior lesion. Potential risk factors include advanced age, pre-existing cervical and lumbar spondylosis, vascular fragility or subclinical atherosclerosis.

In addition to its anatomical rarity, the idiopathic nature of this infarct is also noteworthy. Unlike most reported cases that involve identifiable vascular risk factors such as diabetes, hypertension, or known atherosclerotic disease, our patient had no overt comorbidities (6–9). A comprehensive autoimmune and cardiac workup was unremarkable, further supporting the diagnosis of a cryptogenic, likely microthrombotic, sulcal artery occlusion.

Ultimately, this report adds to the limited but growing body of literature on lumbar spinal cord infarction, particularly of sulcal artery origin, and serves as a reminder to consider vascular events even in the context of routine anaesthetic procedures when patients present with acute focal neurological deficits. This case exemplifies the characteristic presentation of sulcal artery syndrome: unilateral motor weakness with dissociated sensory findings due to hemicord involvement. The key strength in the diagnostic approach was early MRI with dedicated spinal cord sequences, enabling prompt identification of the lesion.

Strengths of this case include early imaging with spinal cord-specific sequences and comprehensive exclusion of mimics such as demyelination or infection. Limitations include the absence of confirmatory angiography, though this is rarely indicated in non-surgical infarcts.

Takeaway Lessons:

• Consider sulcal artery infarction in patients with acute hemicord syndrome, even at the lumbar level.
• Post-spinal anaesthesia neurological deficits warrant prompt MRI to differentiate vascular from compressive or inflammatory causes.
• Idiopathic infarcts may occur in the absence of conventional risk factors, particularly in elderly patients.
• Early diagnosis and rehabilitation are key to improving outcomes in spinal cord infarction.

### Patient perspective

The patient described the onset of symptoms as frightening, particularly the abrupt loss of strength. She expressed gratitude for the clear communication from the medical team and valued the focused rehabilitation. She was optimistic about her recovery and appreciated regaining partial mobility. When contacted six months post the incident, she can walk with a walker and manage her daily activities.

## Conclusions

This case illustrates a rare but significant complication of spinal anesthesia, sulcal artery infarction leading to incomplete Brown-Séquard syndrome. Early neuroimaging, exclusion of reversible causes, and structured rehabilitation are essential to optimizing outcomes. Clinicians must maintain a high index of suspicion in patients with acute neurological deficits after neuraxial procedures.

## Data Availability

The raw data supporting the conclusions of this article will be made available by the authors, without undue reservation.

## Supplementary material

Supplementary Table 1 Clinical timeline of events
