# Supplementary material for: Case Report: Sulcal artery infarction presenting as incomplete Brown-Séquard syndrome following spinal anesthesia in a 70-year-old female: a rare postoperative neurological complication
Source: Front Radiol. 2025 Nov 3;5:1672382. doi: 10.3389/fradi.2025.1672382 (PMC12620253; doi:10.3389/fradi.2025.1672382)
Supplement: Supplementary Table 1 — Clinical timeline of events [file Table1.docx]

**Supplementary Table 1:**

| **Timepoint** | **Clinical Event** |
| --- | --- |
| Day 0 | • Elective hip arthroplasty under spinal anaesthesia  • No intraoperative issues |
| Immediate Post-op | • Sudden onset left lower limb weakness   - Right-sided pain/temperature sensory loss   • Proprioception preserved |
| Day 1 | • Neurological exam consistent with hemicord syndrome |
| Week 4 | • Autoimmune and vascular workup negative |
| Week 5 | - MRI: T2/STIR hyperintensity in left hemicord at D12–L1 - Suggestive of sulcal artery infarct   • No compressive/infective cause on MRI |
| Weeks 5-7 | Initiated supportive care: antiplatelets, DVT prophylaxis, physiotherapy |
| Week 7 | • Gradual neurological improvement  • Mobilising with walker |
| Discharge (Week 8) | • Further improved strength  • Stable condition  •Outpatient rehabilitation advised |
